# Incretin Receptor Agonists and CPAP Use in Adults With Diabetes, Obesity, and Obstructive Sleep Apnea

**DOI:** 10.1001/jamanetworkopen.2025.50978

**Published:** 2025-12-22

**Authors:** Huilin Tang, Bingyu Zhang, Yiwen Lu, Dazheng Zhang, Yuan Lu, David A. Asch, Yong Chen

**Affiliations:** 1 Center for Health AI and Synthesis of Evidence, University of Pennsylvania, Philadelphia; 2 Department of Biostatistics, Epidemiology, and Informatics, University of Pennsylvania Perelman School of Medicine, Philadelphia; 3 The Graduate Group in Applied Mathematics and Computational Science, School of Arts and Sciences, University of Pennsylvania, Philadelphia; 4 Section of Cardiovascular Medicine, Department of Medicine, Yale School of Medicine, New Haven, Connecticut; 5 Center for Outcomes Research and Evaluation, Yale New Haven Health, New Haven, Connecticut; 6 Leonard Davis Institute of Health Economics, University of Pennsylvania, Philadelphia; 7 Wharton School, University of Pennsylvania, Philadelphia; 8 Division of General Internal Medicine, Department of Medicine, Perelman School of Medicine, University of Pennsylvania, Philadelphia; 9 Penn Medicine Center for Evidence-Based Practice, University of Pennsylvania, Philadelphia; 10 Penn Institute for Biomedical Informatics, University of Pennsylvania, Philadelphia, Pennsylvania

## Abstract

This cohort study investigates the association of incretin receptor agonists (IRAs) with continuous positive airway pressure (CPAP) use, mortality, and hospitalization among adults with diabetes, obesity, and obstructive sleep apnea.

## Introduction

Obstructive sleep apnea (OSA) affects approximately 25% of US adults,^1^ frequently coexisting with type 2 diabetes (T2D) due to shared risk factors, such as obesity.^2^ While continuous positive airway pressure (CPAP) remains the first-line treatment for moderate to severe OSA, adherence is often suboptimal, prompting interest in adjunctive strategies that may modify disease severity.^1^ Incretin receptor agonists (IRAs), including glucagon-like peptide-1 receptor agonists (GLP-1RAs) and the dual glucose-dependent insulinotropic polypeptide and GLP-1 RA tirzepatide, have demonstrated substantial weight reduction and metabolic control, while tirzepatide has shown promising effects on OSA severity in recent randomized clinical trials.^3^ However, evidence on their effectiveness in clinical practice remains limited. We evaluated IRA use in routine care among adults with obesity, T2D, and OSA.

## Methods

In this cohort study, we conducted a target trial emulation using the TriNetX research network (January, 2021, to September, 2025), which aggregates deidentified electronic health record (EHR) data from US health care organizations.^4^ Following the target trial protocol (eTable 1 in Supplement 1), we identified 93 193 IRA and 42 534 sodium-glucose cotransporter-2 inhibitor (SGLT2I) initiators among adults (aged ≥18 years) with obesity, T2D, and OSA (eTable 2 in Supplement 1). Outcomes included CPAP use and all-cause mortality and hospitalization. CPAP use was defined using procedure codes (eTable 3 in Supplement 1). Follow-up began at 30 days after treatment initiation and continued until the earliest of outcome, death, 1-year follow-up, or study end. We applied 1:1 propensity score matching (PSM) (nearest-neighbor; caliper 0.1) to balance baseline covariates (standardized mean differences [SMDs] <0.1) (eTable 2 in the Supplement). Baseline covariates were collected within 1 year before treatment initiation. Race and ethnicity were identified from the EHR. Cox proportional hazard models estimated hazard ratios (HRs) with 95% CIs within the matched cohort, with subgroup analyses by age and sex. Further comparisons were conducted between tirzepatide, GLP-1RAs, and SGLT2Is. Analyses were performed using the TriNetX platform and R version 4.5.0 (R Project for Statistical Computing). The study was approved by the University of Pennsylvania Institutional Review Board as exempt from review and consent because data were deidentified and is reported following the STROBE reporting guideline for cohort studies.

## Results

**Figure 1.**
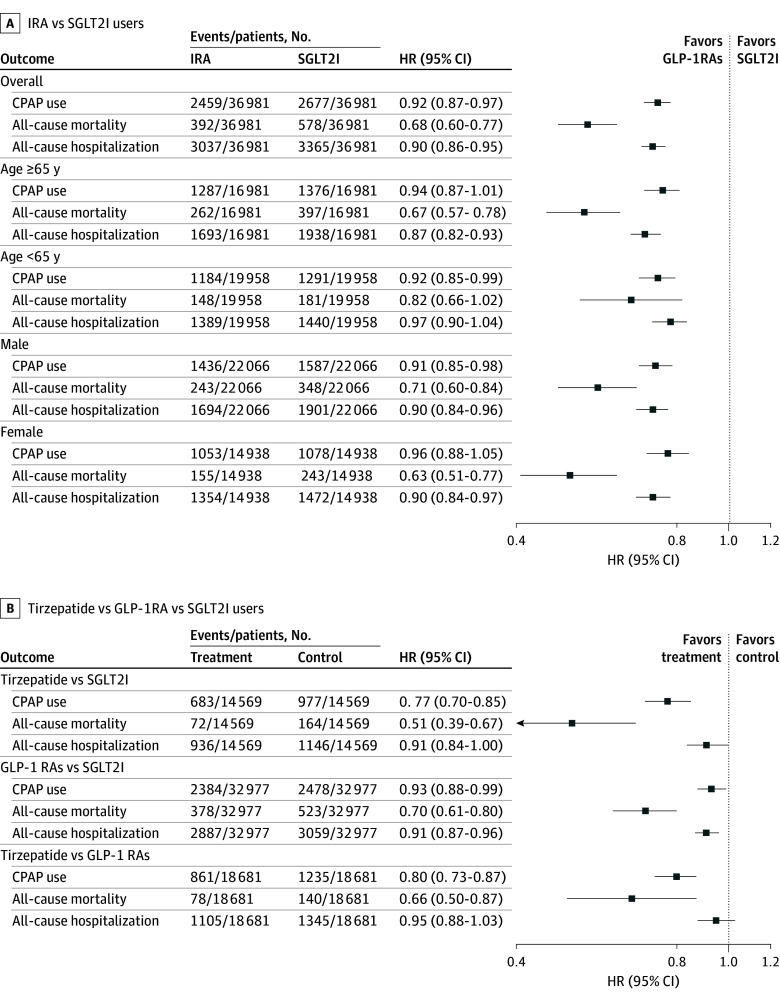
Clinical Outcomes by Drug Type Outcomes are shown comparing incretin receptor agonist (IRA) and SGLT2I users (A) and tirzepatide, glucagon-like peptide-1 receptor agonist (GLP-1RA), and sodium-glucose cotransporter-2 inhibitor (SGLT2I) users (B). CPAP indicates continuous positive airway pressure; HR, hazard ratio.

**Figure 2.**
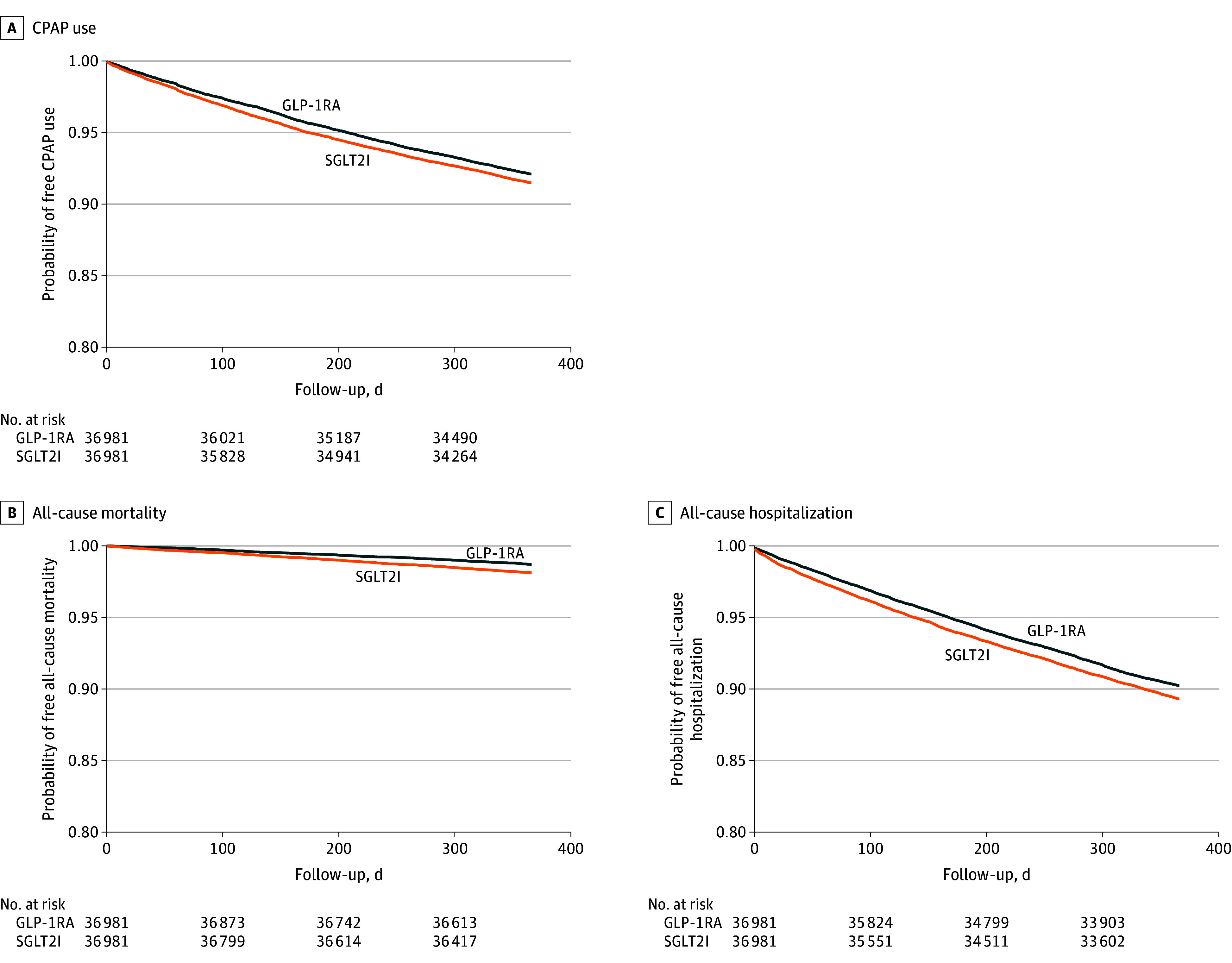
Kaplan-Meier Curves for Clinical Outcomes Curves are shown for continuous positive airway pressure (CPAP) use (A), all-cause mortality (B), and all-cause hospitalization (C) comparing incretin receptor agonist (IRA) and sodium-glucose cotransporter-2 inhibitor (SGLT2I) users.

We identified 36 981 matched pairs of IRA and SGLT2I initiators (mean [SD] age, 62.3 [11.3] years; 39.7% female; 0.6% American Indian or Alaska Native, 2.1% Asian, 18.6% Black, 0.7% Native Hawaiian or Other Pacific Islander, 70.5% White, and 3.5% other race; 6.7% Hispanic) with well-balanced baseline characteristics. The mean (SD) body mass index (calculated as weight in kilograms divided by height in meters squared) was 38.5 (7.2). Over a mean (SD) follow-up of 332.2 (116.3) days, 2459 IRA (6.6%) and 2677 SGLT2I (7.2%) users received CPAP therapy. Compared with SGLT2I use, IRA use was associated with decreased risks of CPAP use (HR, 0.92; 95% CI, 0.87-0.97) and all-cause mortality (HR, 0.68; 95% CI, 0.60-0.77) and hospitalization (HR, 0.90; 95% CI, 0.86-0.95) (Figure 1; Figure 2). Subgroup analyses showed broadly consistent results, although no association was found for CPAP use in females or older adults. Tirzepatide and GLP-1RAs were associated with decreased risks of CPAP use and mortality compared with SGLT2Is, with tirzepatide showing greater decreases than GLP-1RAs (Figure 1).

## Discussion

In adults with obesity, T2D, and OSA in this cohort study, initiation of IRAs, particularly tirzepatide, was associated with decreased risk of CPAP use, hospitalization, and mortality compared with SGLT2Is. These findings align with recent randomized clinical trials^3,5^ showing that IRAs, especially tirzepatide, improved OSA severity, potentially through weight loss and metabolic or respiratory mechanisms. The modest effect size for IRAs suggests limited clinical relevance for CPAP use alone, but reductions in mortality and hospitalization support broader cardiometabolic benefits. Notably, tirzepatide showed greater reductions in risk of CPAP use and mortality than GLP-1RAs, highlighting its potential therapeutic advantage. The attenuated association among females may reflect sex-related differences in OSA pathophysiology given that males typically have higher OSA severity.^6^ Limitations include that procedural records for CPAP may not reflect actual use, adherence, or OSA severity and that unmeasured confounding, such as socioeconomic status and OSA severity (eg, apnea-hypopnea index), may influence the observed association. Future research should clarify causal pathways linking pharmacologic weight loss with OSA outcomes.

## Notes

Supplement 1. **eTable 1.** Target trials emulation **eTable 2.** Baseline characteristics of patients in incretin receptor agonist (IRA) and SGLT2I groups **eTable 3.** Diagnostic, outcome, and drug codes

Supplement 2.
Data Sharing Statement
