# Supplementary material for: Incretin Receptor Agonists and CPAP Use in Adults With Diabetes, Obesity, and Obstructive Sleep Apnea
Source: JAMA Netw Open. 2025 Dec 22;8(12):e2550978. doi: 10.1001/jamanetworkopen.2025.50978 (PMC12723546; doi:10.1001/jamanetworkopen.2025.50978)
Supplement: Supplement 1. — eTable 1. Target trials emulation eTable 2. Baseline characteristics of patients in incretin receptor agonist (IRA) and SGLT2I groups eTable 3. Diagnostic, outcome, and drug codes [file jamanetwopen-e2550978-s001.pdf]

Supplemental Online Content

Tang H, Zhang B, Lu Y, et al. Incretin receptor agonists and CPAP use in adults with diabetes, obesity, and obstructive sleep apnea. *JAMA Netw Open*. 2025;8(12):e2550978. doi:10.1001/jamanetworkopen.2025.50978

eTable 1. Target trials emulation

eTable 2. Baseline characteristics of patients in incretin receptor agonist (IRA) and SGLT2I groups

eTable 3. Diagnostic, outcome, and drug codes

This supplemental material has been provided by the authors to give readers additional information about their work.

© 2025 Tang H et al. *JAMA Network Open*.

Table S1. Target trial emulation

Approaches	Target trial	Target Trial Emulation
Eligibility criteria	● Obese adults with type 2 diabetes and obstructive sleep apnea<br>● Age≥ 18 years<br>● No diagnosis of type 1 diabetes<br>● No prior use of incretin receptor agonist or SGLT2I<br>● No contraindication to study drugs (end-stage renal disease/dialysis)<br>● No prior outcomes of interest	Same
Treatment strategies	Initiation treatment with an incretin receptor agonist, or SGLT2I	The date of initiation treatment was the date of the first prescription of an incretin receptor agonist or SGLT2I
Treatment assignment	Individuals are randomly assigned to incretin receptor agonist or SGLT2I at baseline. Individuals and their treating physicians will be aware of the assigned treatment strategy.	Randomization was emulated using 1:1 propensity score matching to address potential confounders.
Outcomes	CPAP use, all-cause mortality, and all cause hospitalization	Same
Follow-up	Follow-up began at 30 days after treatment initiation and continued until the earliest of outcome, death, 1 year follow-up, or study end.	Same
Causal contrasts	Intention-to-treat effect	Same
Statistical analysis	Cox proportional hazard regression models and Kaplan–Meier survival plot. Subgroup analyses would be performed by age (≥ 65 vs. <65 years), sex (female vs. male) and between tirzepatide, GLP-1RAs, and SGLT2Is.	Same

CPAP, continuous positive airway pressure; GLP-1RAs, glucagon-like peptide-1 receptor agonists; SGLT2Is, sodium-glucose cotransporter-2 inhibitors

© 2025 Tang H et al. *JAMA Network Open*.

Table S2. Baseline characteristics of patients in incretin receptor agonist (IRA) and SGLT2I groups

**Characteristic**	**Before 1:1 PSM**			**After 1:1 PSM**		
	**IRA (n=93193)**	**SGLT2I (n=42534)**	**SMD**	**IRA (n=36981)**	**SGLT2I (n=36981)**	**SMD**
Age at Index	58.03(12.24)	63.22(11.62)	0.435	62.42(11.09)	62.26(11.56)	0.014
**Sex**						
Female	50.13%	37.77%	0.251	39.48%	39.85%	0.007
Male	49.85%	62.21%	0.251	60.49%	60.14%	0.007
Unknown Gender	0.02%	0.03%	0.004	0.03%	0.03%	0.003
**Ethnicity**						
Hispanic or Latino	7.18%	6.87%	0.012	6.60%	6.78%	0.007
Not Hispanic or Latino	76.10%	75.81%	0.007	76.00%	75.92%	0.002
Unknown Ethnicity	16.71%	17.32%	0.016	17.40%	17.31%	0.003
**Race**						
White	70.30%	69.94%	0.008	70.62%	70.28%	0.007
Black or African American	18.80%	19.05%	0.006	18.56%	18.70%	0.004
Asian	1.94%	2.09%	0.011	2.13%	2.13%	0.000
Native Hawaiian or Other Pacific Islander	0.73%	0.69%	0.005	0.66%	0.68%	0.002
American Indian or Alaska Native	0.64%	0.60%	0.004	0.55%	0.59%	0.006
Other Race	3.42%	3.54%	0.007	3.45%	3.51%	0.003
Unknown Race	4.18%	4.08%	0.005	4.04%	4.11%	0.004
**Comorbidities**						
Diabetic nephropathy	12.77%	24.15%	0.296	21.10%	21.04%	0.001
Diabetic neuropathy	14.89%	19.86%	0.132	19.37%	19.07%	0.008
Diabetes circulatory complications	7.43%	10.34%	0.102	9.73%	9.62%	0.004
Diabetic retinopathy	4.72%	6.49%	0.077	6.31%	6.18%	0.005
Cerebrovascular diseases	5.22%	8.94%	0.145	7.83%	7.92%	0.003
Hypertensive diseases	81.06%	88.65%	0.213	87.87%	87.54%	0.010

© 2025 Tang H et al. *JAMA Network Open*.

Lipid disorders	73.81%	80.42%	0.158	80.24%	79.59%	0.016
Ischemic heart diseases	18.43%	35.30%	0.388	29.99%	30.46%	0.010
Atrial fibrillation and flutter	9.72%	20.57%	0.306	16.48%	16.72%	0.006
Heart failure	10.29%	31.90%	0.549	22.80%	23.79%	0.024
Liver disease	14.37%	12.02%	0.069	12.41%	12.39%	0.001
Mood disorders	30.27%	24.68%	0.125	25.39%	25.64%	0.006
Anxiety	28.66%	22.45%	0.143	23.31%	23.31%	0.000
Thyroid disease	21.39%	20.60%	0.019	20.58%	20.60%	0.001
Vitamin D deficiency	22.27%	17.88%	0.110	18.49%	18.63%	0.003
Substance use disorders	12.74%	14.26%	0.044	13.78%	13.75%	0.001
Headache	7.08%	5.90%	0.048	6.08%	6.11%	0.001
Migraine	6.77%	4.25%	0.110	4.70%	4.62%	0.004
Neoplasms	23.09%	23.38%	0.007	23.90%	23.48%	0.010
**Comedications**						
Metformin	47.78%	47.56%	0.004	49.70%	48.88%	0.016
Insulins and analogues	21.77%	32.08%	0.234	29.85%	29.69%	0.004
Dipeptidyl peptidase 4 inhibitors	5.44%	9.17%	0.143	8.79%	8.75%	0.001
Sulfonylureas	12.44%	16.97%	0.128	17.19%	16.60%	0.016
Thiazolidinediones	2.51%	3.44%	0.054	3.58%	3.51%	0.004
Agents acting on the renin-angiotensin system	49.87%	60.66%	0.218	58.94%	58.58%	0.007
Diuretics	38.10%	51.74%	0.277	47.35%	47.57%	0.004
Beta blocking agents	31.76%	49.13%	0.359	44.15%	44.28%	0.003
Calcium channel blockers	24.86%	31.20%	0.142	30.13%	30.20%	0.001
Lipid modifying agents	56.38%	67.92%	0.240	66.55%	65.97%	0.012
Other analgesics and antipyretics	42.43%	53.45%	0.222	50.67%	50.40%	0.005
Corticosteroids for systemic use	41.56%	40.07%	0.030	39.97%	39.93%	0.001
Antithrombotic agents	24.93%	44.09%	0.412	38.62%	38.70%	0.002

© 2025 Tang H et al. *JAMA Network Open*.

Antidepressants	35.63%	32%	0.077	32.44%	32.51%	0.002
Opioids	27.00%	33.83%	0.149	31.65%	31.77%	0.002
Antiinflammatory and antirheumatic products	31.58%	27.37%	0.092	28.35%	28.31%	0.001
Antiepileptics	25.13%	26.84%	0.039	26.85%	26.40%	0.010
Hypnotics and sedatives	19.85%	26.12%	0.149	23.70%	23.89%	0.005
Thyroid therapy	13.37%	13.26%	0.003	13.19%	13.21%	0.001
Antihypertensives	8.43%	13.75%	0.170	11.84%	12.13%	0.009
Antipsychotics	9.83%	10.46%	0.021	10.20%	10.26%	0.002
Antineoplastic agents	6.63%	6.32%	0.012	6.44%	6.52%	0.003
Anti-parkinson drugs	4.06%	4.58%	0.026	4.51%	4.50%	0.001
Hormones and related agents	1.66%	0.75%	0.083	0.71%	0.79%	0.008
Anti-obesity medication	1.90%	0.44%	0.136	0.43%	0.50%	0.012
**Lab and vital values**						
SBP, mmHg, mean(sd)	130.81(15.84)	129.63(18.00)	0.070	130.78(16.43)	130.00(17.62)	0.046
≥ 130	76.66%	76.52%	0.003	76.56%	76.64%	0.002
DBP, mmHg, mean(sd)	77.59(10.67)	75.21(11.98)	0.210	76.27(10.85)	75.68(11.85)	0.052
≥ 90	35.49%	32.92%	0.054	32.78%	32.99%	0.004
BMI, kg/m^2	40.72(7.81)	37.82(7.03)	0.390	38.79(7.32)	38.20(7.12)	0.081
30-35	25.28%	38.08%	0.278	35.47%	35.65%	0.004
35-40	31.52%	33.36%	0.039	33.53%	33.21%	0.007
≥ 40	43.78%	31.19%	0.262	33.03%	33.10%	0.002
eGFR, mL/min/1.73m^{2,} mean(sd)	79.99(25.14)	72.26(26.57)	0.299	74.45(25.35)	74.01(26.42)	0.017
≥ 45	78.12%	75.87%	0.053	76.34%	76.13%	0.005
HbA1c,%, mean(sd)	7.49(1.72)	7.82(1.71)	0.190	7.71(1.73)	7.84(1.71)	0.080
≥ 7 %	44.45%	50.25%	0.116	52.26%	50.50%	0.035
HDL, mg/dL, mean(sd)	41.47(15.10)	39.55(15.29)	0.126	40.83(15.03)	39.74(15.19)	0.072

© 2025 Tang H et al. *JAMA Network Open*.

< 40	31.86%	32.71%	0.018	32.97%	32.63%	0.007
LDL, mg/dL, mean(sd)	90.36(36.09)	81.67(35.48)	0.243	82.12(34.33)	83.11(35.81)	0.028
≥ 100	27.39%	18.51%	0.212	19.48%	19.79%	0.008
Triglyceride, mg/dL, mean(sd)	181.02(145.85)	186.94(177.85)	0.036	181.08(149.40)	190.56(179.74)	0.057
≥ 150	36.14%	33.03%	0.065	34.71%	34.36%	0.007
Cholesterol, mg/dL, mean(sd)	166.76(43.46)	157.27(45.74)	0.213	157.74(42.38)	159.44(46.08)	0.038
≥ 200	16.49%	11.48%	0.145	12.19%	12.32%	0.004

IRA, incretin receptor agonist; SGLT2I, sodium-glucose cotransporter-2 inhibitor;
PSM, propensity score matching; SMD, standardized mean difference; SD, standard deviation; DBP, diastolic blood pressure; SBP, systolic blood pressure; BMI, body mass index; eGFR, estimated glomerular filtration rate; LDL, low-density lipoprotein; HDL, high-density lipoprotein; HbA1c, glycated hemoglobin.

© 2025 Tang H et al. *JAMA Network Open*.

Table S3. Diagnostic, outcome, and drug codes

**Category**	**Diagnosis, outcomes and drugs**	**Codes**
Outcomes	CPAP use	ICD-10-CM: Z99.89<br>ICD-10-PCS: 5A09357<br>CPT: 94660<br>HCPCS: E0601
	All-cause hospitalization	CPT: 1013659
	All-cause mortality	Deceased;<br>ICD-10-CM: R99
Incretin receptor agonist	GLP-1RA	ATC: A10BJ
	Tirzepatide	RxNorm: 2601723
SGLT2I	SGLT2I	ATC: A10BK
Inclusion/ exclusion criteria	Obesity	Body mass index >30 kg/m^2 or<br>ICD-10-CM: E66.0, E66.1, E66.2, E66.8, E66.9, Z68.3, Z68.4
	Type 2 diabetes	ICD-10-CM: E11
	End stage kidney disease/dialysis	ICD-10-CM: N18.6, Z99.2
	Type 1 diabetes	ICD-10-CM: E10

GLP-1RA, glucagon-like peptide-1 receptor agonist; SGLT2I, sodium-glucose cotransporter-2 inhibitor; CPAP, continuous positive airway pressure; ICD-10-CM, the International Classification of Diseases, Tenth Revision, Clinical Modification; ATC, Anatomical Therapeutic Chemical Classification System; CPT, Current Procedural Terminology; HCPCS, Healthcare Common Procedure Coding System

© 2025 Tang H et al. *JAMA Network Open*.
