# Supplementary material for: Incretin Receptor Agonists and CPAP Use in Adults With Diabetes, Obesity, and Obstructive Sleep Apnea
Source: JAMA Netw Open. 2025 Dec 22;8(12):e2550978. doi: 10.1001/jamanetworkopen.2025.50978 (PMC12723546; doi:10.1001/jamanetworkopen.2025.50978)
Supplement: Supplement 2. — Data Sharing Statement [file jamanetwopen-e2550978-s002.pdf]

Data Sharing Statement

Tang. Incretin Receptor Agonists and CPAP Use in Adults With Diabetes, Obesity, and Obstructive Sleep Apnea. *JAMA Netw Open*. Published online December 22, 2025.
doi:10.1001/jamanetworkopen.2025.50978

Data
Data available: No

Additional Information
Explanation for why data not available: This study used population-level aggregate and de-identified data generated by the TriNetX platform. The data that support the findings of this study are available from the TriNetX Analytics Network. https://trinetx.com.
